# Pharmacogenomic markers of glucocorticoid response in congenital adrenal hyperplasia

**DOI:** 10.1371/journal.pone.0279298

**Published:** 2022-12-20

**Authors:** Cristina Botelho Barra, Thais Ramos Villela, Nedstâni de Freitas Soares, Enrico Antônio Colosimo, André Rolim Belisário, Ana Cristina Simões e Silva, Ivani Novato Silva

**Affiliations:** 1 Department of Pediatrics, Faculty of Medicine, Universidade Federal de Minas Gerais (UFMG), Belo Horizonte, Minas Gerais, Brazil; 2 Pediatric Endocrinology Division, Hospital das Clínicas da Universidade Federal de Minas Gerais (UFMG), Belo Horizonte, Minas Gerais, Brazil; 3 Department of Statistics, Universidade Federal de Minas Gerais (UFMG), Belo Horizonte, Minas Gerais, Brazil; 4 Interdisciplinary Laboratory of Medical Investigation, Faculty of Medicine, Universidade Federal de Minas Gerais (UFMG), Belo Horizonte, Minas Gerais, Brazil; Osaka City General Hospital, Children’s Medical Center, JAPAN

## Abstract

Glucocorticoids (GC) replacement are the mainstay treatment for 21-hydroxylase deficiency (21-OHD), the most common cause of congenital adrenal hyperplasia (CAH), in its classical form. There are novel insights into the genetic basis of the GC action diversity that point to an important role for GC receptor (GR) gene polymorphisms, suggesting a possible modulation in occurrence of metabolic disorders, what may be relevant to clinical management of 21-OHD. The aim of this study was to investigate whether the five GR gene polymorphisms *Tth111*I, *ER22*, *23EK*, *Bcl*I, *9β* (rs10052957, rs6189, rs6190, rs41423247, rs6198) and their combination into haplotypes are associated to different GC response in a cohort of classic 21-OHD subjects. GR genotype-phenotype associations were explored after a dexamethasone suppression test using very low-doses (VLD-DST), 20 and 40 μg/m². The final sample (n = 28) was selected based on the 102 individuals’ previous genotypes classification, according to literature data of GC sensitivity or resistance. Thus, only patients with GC increased resistance (n = 18) or increased sensitivity (n = 10) profiles were selected. Out of 28 subjects aged 12 (2–34) years enrolled in this study, 75% were females, 75% presented the salt-wasting form (SW) and 25% the simple virilizing form (SV). Subjects who carried *Tth111*I and *9β*, associated or not to the *ER22/23EK* variants, showed an impaired DST response. Results did not differ significantly according to gender or body mass index. SV subjects with GC hypersensitivity-genotypes showed decreased average cortisol levels compared to those with GC resistance-genotypes (p = 0.0023). The *Tth111*I *+ 9β/ Wild* or *Tth111*I *+ ER22/23EK + 9β/ Wild* genotypes were associated to GC resistance in this population. This finding may be relevant given the challenges posed by therapeutic management with GC in CAH.

## Introduction

Glucocorticoids (GC) replacement are the mainstay treatment for 21-hydroxylase deficiency (21-OHD), the most common cause of congenital adrenal hyperplasia (CAH), a group of autosomal recessive disorders affecting cortisol biosynthesis [1]. The inability to restore physiological cortisol secretion rhythm compromises the outcomes [2, 3]. Therefore, many challenges remain for the management of these patients [4].

The variety in clinical responses to treatment with GC reflects the variation in GC sensitivity between individuals; while some patients are known to rapidly develop adverse effects during corticotherapy, others show good tolerance. Furthermore, reduced glucocorticoid sensitivity has been associated to more favourable metabolic profile, while glucocorticoid hypersensitivity might be involved in the pathogenesis of the metabolic syndrome and mood disorders. Genetic factors that are associated to GC sensitivity are involved in the individuals’ response to GC and the predisposition for diseases [5].

The GC response and the pituitary negative feedback are regulated through GC binding to its receptor (GR), encoded by the *NR3C1* gene. Differences in healthy individuals’ response to GC are, at least in part, genetically determined by the *NR3C1* gene polymorphisms [5, 6].

Novel insights into the basis of the GC sensitivity point to an important role for GR gene variants [7–10]. Two reports suggest that polymorphisms of the GR gene may be associated with metabolic profiles in 21-OHD patients: the *Bcl*I GR polymorphism, which is associated to increased GC sensitivity, was linked to increased cardiovascular risk among adult CAH-subjects [11]. Conversely, the *9β*-variant, which was associated to healthier metabolic profiles among paediatric subjects with CAH, is expected to increase GC resistance [12].

Specific arrangements of the *NR3C1* gene [13] polymorphisms may play a role in the interindividual variability to GC treatment of 21-OHD subjects. We have investigated whether single nucleotide polymorphisms (SNPs) previously linked to GC increased resistance or sensitivity were associated to different GC responses in a cohort of 21-OHD subjects.

## Methods

We have investigated six SNPs of the GR gene, estimated each minor allelic frequency (MAF) and the haplotypes in CAH-subjects (n = 102). The comparative analysis of MAF with a control group of healthy subjects (n = 163) was previously studied [14] and is shown in **Table 1**.

**Table 1:** Minor allelic frequencies (MAF) of the studied SNPs: *Tth111*I (rs10052957), *ER22*/*23EK* (rs6189, rs6190), *N363S* (rs56149945), *Bcl*I (rs41423247) and *9β* (rs6198) in 102 CAH-subjects and 163 healthy controls.

*NR3C1* Gene Polymorphisms	Cases (n = 102)	Controls (n = 163)
***Tth111*I**	32.8%	25.8%
***ER22/ 23EK***	2.5%	3.3%
***N363S***	0.5%	1.7%
***Bcl*I**	26%	32.7%
***9β***	16.2%	11.6%

[ref 14]

**Fig 1:**
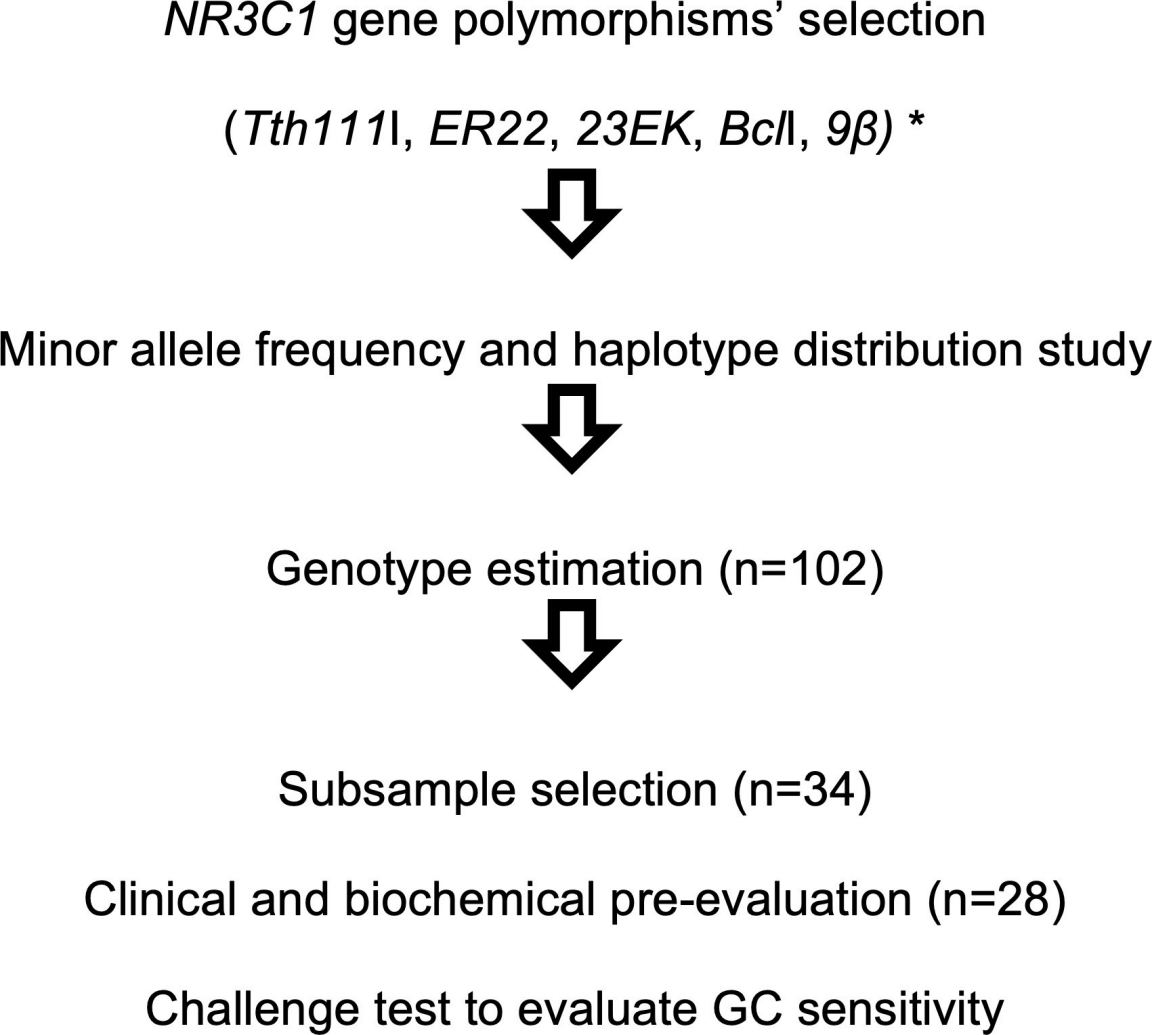
Study flowchart: Step 1: Literature reviewing on the most studied *NR3C1* polymorphisms; step 2: Determining the frequencies of minor alleles and haplotypes; step 3: Estimation of genotypes; step 4: Selection of genotypes with greater sensitivity or greater resistance to GC, for genotype-phenotype association study among subjects with CAH. *(SNPs ID: rs10052957, rs6189, rs6190, rs41423247, rs6198). CAH: congenital adrenal hyperplasia; GC: glucocorticoid.

GR genotype-phenotype associations were, then, explored after a very-low dose dexamethasone suppression test (VLD-DST). The study complete flowchart is shown in **Fig 1**.

### Ethics

The study was approved by the Ethics Committee of the Institution (CAAE-1.172.019). Patients and their legal guardians signed an informed written consent.

### Participants

The population of interest were classic 21-OHD patients under GC replacement and regular monitoring at the Federal University of Minas Gerais, Hospital of Clinics (HC-UFMG). The 21-OHD classic form diagnosis was based on clinical and biochemical evaluation, according to medical records. Salt-wasting (SW) subjects who presented well-documented hyponatremia and hyperpotassaemia in the neonatal period were selected; some SW adult patients were not on fludrocortisone replacement. Simple virilizing (SV) ones were diagnosed by the public neonatal screening program of the state of Minas Gerais and were not on fludrocortisone replacement. Patients with either chronic disease or taking medication other than hormone replacement for CAH had not been included. A sample of 102 unrelated subjects with a median of 8.95 years-old [interquartile range (IQR) 2.13–17.95], 73 females (71.6%) was enrolled in the GR-genotyping study.

### *NR3C1* genotyping

**Fig 2:**
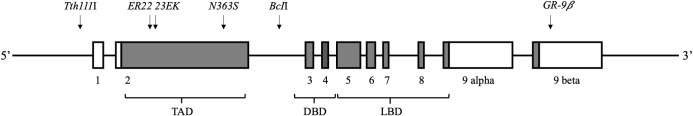
Schematic representation and localization of the *NR3C1* gene polymorphisms *Tth111*I, *ER22*, *23EK*, *N363S*, *Bcl*I and *9β* (rs10052957, rs6189, rs6190, rs56149945, rs41423247, rs6198). *Tth111*I: in flanking area; *ER22*, *23EK*, *N363S*: exon 2 at TAD; *Bcl*I: intron between exons 2 and 3; 9β: exon 9. TAD: transactivation domain; DBD: DNA binding domain; LBD: ligand binding domain.

The human GR gene (*NR3C1*) is located within a single linkage disequilibrium block in chromosome 5 (5q31.3). It spans a length of 157,582 bases and is comprised of 13 first non-coding exons, which act as alternative promoters, and 8 protein-coding regions or exons numbered 2–9 [13]. Among the several SNPs of this gene, the ones linked to the GC response were studied here: *Tth111*I, *ER22*, *23EK* and *9β are associated to GC resistance*, *while* N*363S* and *Bcl*I are associated to GC increased sensitivity (**Fig 2**). Genotyping methods were described elsewhere [14].

### Clinical assessment

The VLD-DST final sample (n = 28) was selected based on the 102 individuals’ genotypes classification, according to data of GC sensitivity or resistance of the GR gene variants, well stated in previous reports [8]. Thus, only patients with GC increased resistance (n = 18) or increased sensitivity (n = 10) profiles were selected. Increased resistance or sensitivity were assumed by selecting the genotypes with 100% likelihood estimation of GC resistance or sensitivity in the haplotype distribution of this population.

Subjects with 21-OHD, who were in good health conditions, were recruited to undergo the VLD-DST when serum 17-hydroxyprogesterone (17OHP) was > 1,000 ng/dL (by immune chemiluminometric assay) on the eve of the test [2]. After consent, the GC in use was discontinued according to the respective biological half-life of each drug: 4 days for hydrocortisone, 6 days for prednisone and 7 days for dexamethasone [15, 16]. There was no alteration in the administration schedule of fludrocortisone, given once daily in the morning. After GC withdrawal, participants were carefully monitored by phone; they were instructed to inform any symptoms of disease and to resume glucocorticoid use immediately.

A single trained observer obtained standard anthropometric measurements with the patients wearing appropriate clothes. Body mass index (BMI) was calculated by [weight (kg)/height (m)^2^] and body surface area (BSA) was calculated by [weight (kg) x 4 + 7/90 + weight (kg)] [17].

### Very low-dose dexamethasone suppression test protocol

The VLD-DST test was used to primarily access the GC negative feedback at the level of the anterior pituitary as it only partially suppresses cortisol levels [15, 16]. Standard VLD-DST protocols [18] were adapted to assess individual GC sensitivity for this population.

Adrenocorticotropic hormone (ACTH) levels are intricately entwined with cortisol levels, even in CAH-subjects [32]. Here, cortisol decreased concentrations along the test were considered as a suppressive test response. Participants who did not present any decline in cortisol levels, were called as non-suppressors.

The test was carried out at HC-UFMG Laboratory Medical Service. Serum cortisol (without fasting) was measured at 8:00 (baseline) and after two and four hours after very low doses (20 and 40 μg/m²) of intravenous dexamethasone disodium phosphate in bolus (Decadron® 2mg/mL, Aché Laboratórios Farmacêuticos S.A., Brazil). Venepuncture was performed by an experienced nurse, who also prepared the solution: 1 mL of the product diluted in 19 mL of 0.9% saline solution to a final concentration of 100 μg/mL.

### Serum cortisol assay

Blood samples were analysed in the same institution for serum cortisol, by a competitive chemiluminescent enzyme immunoassay on the Integrated VITROS™ 5600 Microwell platform (Johnson & Johnson, High Wycombe, UK, 2009). The manufacturer’s range for serum basal cortisol is 4.46–22.7 μg/dL. The provided detection limit (sensitivity) is > 0.1 μg/dL and the working range is 0.16–61.6 μg/dL. The cortisol mean cross-reactivity with biological steroid precursors is <0.5%. The 20 μg/m²-DST intraindividual cortisol variability in healthy volunteers was 4.3% in a previous study [18].

### Statistical analysis

The genotypes were estimated computationally by the Haplo.stats package (1.6.3 version), available in R, 3.5.1 version. This software uses a consistent maximum likelihood estimation algorithm (haplo-em function) and calculates specific values for the haplotypes (haplo-score function), considering p<0.05, and further estimates carrier probability assuming a diallelic model of inheritance. Cases eligible for the genotype-phenotype association study were selected by assuming this population haplotype distribution and then, selecting only genotypes with 100% likelihood estimation.

GC sensitivity trough the VLD-DST was studied according to age (<10, 10–20 or > 20 years old), designated sex (female or male), CAH clinical form (SW or SV), the current use of fludrocortisone (among SW), and GC dose. For uniform analysis, long-acting GC were transformed into equivalent doses of hydrocortisone, as follows: prednisone: hydrocortisone mg x 4; dexamethasone: hydrocortisone mg x 30 [17]. The non-parametric Kruskal-Wallis (KW) test was used to compare medians and chi-square (x^2^) statistic for mean values.

Baseline and suppressed by dexamethasone serum cortisol (F) values were evaluated together as continuous variables (average cortisol values) and as percentage changes. Outlier evaluation followed the limit of 1.5 times the interquartile range (IQR). Multiple regression analysis was performed using a random effect model for average cortisol values.

The analysis was performed using R (version 3.6.1, Core Team, 2019), Minitab Statistical (version 17.1.0, 2010) and Microsoft® Excel for Mac (version 16.32, 2019) Software. Two time-effect models were built to study the average cortisol levels across the test compared to baseline for SV and SW subjects. Statistical significance was set as p<0.05.

## Results

The subjects’ clinical features are summarized in **Table 2** and their genotype distribution in **Table 3** (n = 28). An outlier (male SW-child) was excluded from analysis.

**Table 2:** Clinical characteristics of 21-OHD subjects (n = 28).

Characteristics	n (%)
**Age-groups (full years old)** ^a^	
<10	9 (32%)
10–20	9 (32%)
>20	10 (36%)
**Biological sex**	
Female	21 (75%)
Male	7 (25%)
**21-OHD classical form**	
Salt-wasting	21 (75%) (5 children, 7 adolescents, 9 adults)
Simple virilizing	7 (25%) (4 children, 2 adolescents, 1 adult)
**Fludrocortisone use**	
Salt-wasting	16/21 (76%)
Simple virilizing	0
**Long-term GC (type)**	
Hydrocortisone	16 (57%) (10 children, 6 adolescents)
Prednisone	9 (32%) (2 adolescents, 7 adults)
Dexamethasone	3 (11%) (all adults)
**Long-term GC (dose in mg/m**^**2**^**)** ^b^	
Median	11.5
IQR	8–12.5
**BMI status**	
Eutrophic	15 (54%)
Overweight or obese	13 (46%)

^a^ <10: children, 10–20: adolescents, >20: adults

^b^ hydrocortisone-equivalent dosing

21-OHD: 21-hydroxylase deficiency; GC: glucocorticoid; IQR: interquartile range; BMI: body mass index

**Table 3:** 1) All genotypes (n = 102). 2) genotypes from final sample, listed according to GC increased resistance (A) or increased sensitivity (B) patterns in 21-OHD subjects (n = 28).

**1.**	
**Genotypes**	**n**
W/W	29
*Bcl*I + *Tth111*I/ W	19
*Tth111*I + *9**β***/ W	17
*Bcl*I/ W	10
*Tth111*I/ W	5
*Tth111*I + *9**β**/ Bcl*I*	5
*Bcl*I + *Tth111*I/ *Bcl*I	4
*Bcl*I + *Tth111*I/ *9**β*** + ER22 + 23EK*	3
*Bcl*I/ *Bcl*I	2
*Tth111*I + *9**β***/ *Tth111*I + *9**β*** + *ER22* + *23EK*	2
*Bcl*I + *Tth111*I/ *Bcl*I + *Tth111*I	1
W/ *Tth111*I + *9**β*** + *ER22* + *23EK*	1
*Tth111*I/ *Tth111*I + *9**β*** + *ER22* + *23EK*	1
*Bcl*I/ *Tth111*I + *9**β*** + *ER22* + *23EK*	1
*Tth111*I + *Bcl*I/ *Tth111*I + *Bcl*I + *9**β***	1
*N363S*/ W	1
	102
**2.**	
A. Selected Genotypes of Increased Resistance	
*Tth111*I + *9**β***/ W	15
*Tth111*I + *9**β***/ *Tth111*I + *9**β*** + *ER22* + *23EK*	1
*Tth111*I/ *Tth111*I + *9**β*** + *ER22* + *23EK*	1
W/ *Tth111*I + *9**β*** + *ER22* + *23EK*	1
	18
B. Selected Genotypes of Increased Sensitivity	
*Bcl*I/ W	7
*Bcl*I/ *Bcl*I	2
*N363S*/ W	1
	10

21-OHD: 21-hydroxylase deficiency; GC: glucocorticoid

Baseline cortisol concentrations were lower among non-suppressors, compared to suppressors (p<0.0039, KW) and were not statistically different between children, adolescents, and adults.

**Fig 3:**
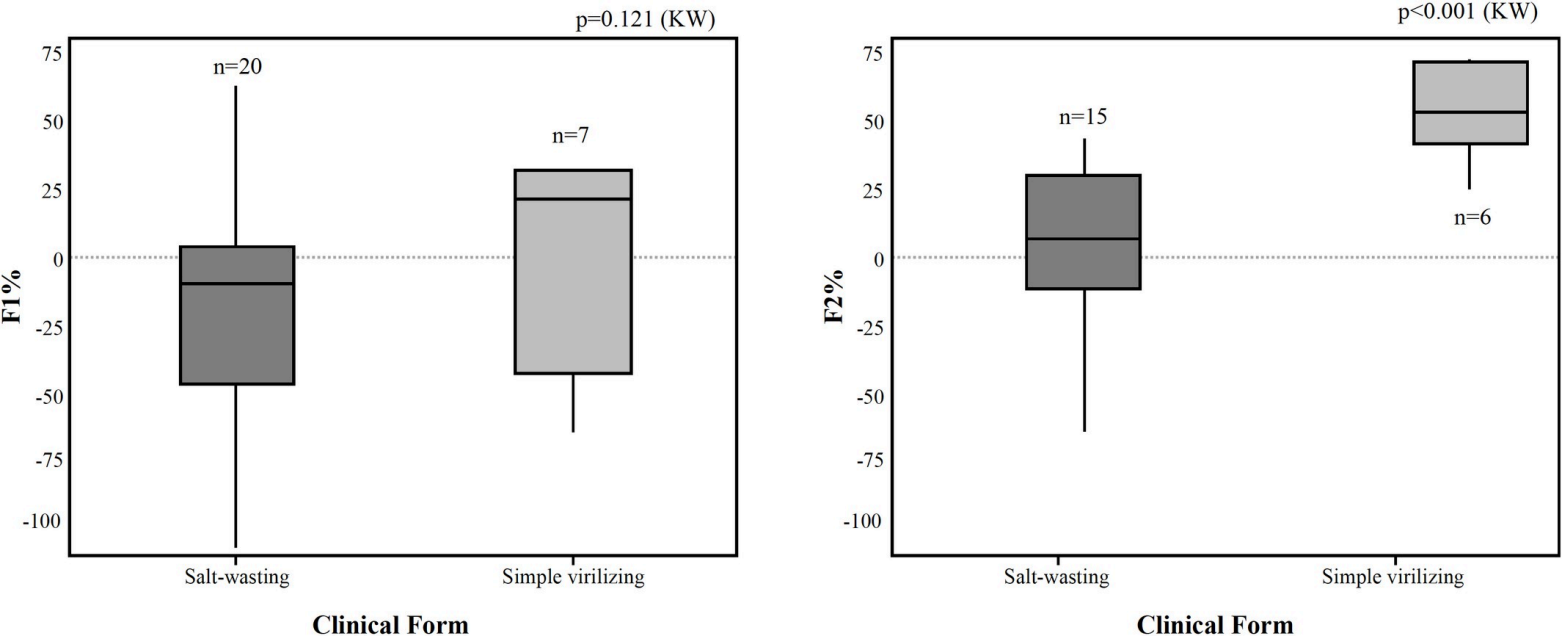
Boxplots. Distribution of dexamethasone suppressed cortisol (F1% and F2%) of 21-OHD-subjects according to their clinical form. n = 27. Any percentage reduction was considered a positive suppressive response. F1% = 100 - (F_2 hours_ x 100/F _basal_); F2% = 100 - (F_4 hours_ x 100)/F_2 hours_); 21-OHD: 21-hydroxylase.

The clinical variables biological sex, GC use (type and dose), and BMI did not affect the GC response. SW subjects presented less cortisol suppression compared to SV ones (**Fig 3**).

The *Tth111*I *+ 9β/ Wild* or *Tth111*I *+ ER22/23EK + 9β/ Wild* genotypes were associated to GC resistance. Six participants had no cortisol suppression at all (**Table 4**).

**Table 4:** Pharmacogenomic markers of glucocorticoid resistance in 21-OHD subjects (n = 6).

Genotype	DST non-suppressors
*Tth111*I *+ 9β/ W*	5 (83%)
*Tth111*I *+ ER22/23EK + 9β/ W*	1 (17%)

21-OHD: 21-hydroxylase deficiency; DST: dexamethasone suppression test; W: wild.

Non-suppressors were all SW subjects with median 17OHP of 3448 ng/dL (ranging 1443 to 10,000 ng/dL) on the eve of the challenge test. They did not differ from suppressors for GC replacement dose (p = 0.202, x^2^).

The most significant cortisol suppression was observed in a homozygous *Bcl*I-carrier. No significant differences were observed at 2-hour testing (p = 0.121) in the comparison of cortisol variability between SW and SV subjects. However, significant differences were detected at 4-hour testing (p<0.001). These significant differences remained when data of fludrocortisone non-users (SW and SV subjects) were compared (p = 0.038, KW).

The two time-effect models for cortisol suppression between SW and SV subjects are presented in **Table 5**. SV subjects exhibited a statistically significant decrease in cortisol levels at the 4-hour time-point of the test (p = 0.0064). The final multivariate model for SV subjects included age (p = 0.0155) and genotype (p = 0.0023). Subjects with GC increased sensitivity-genotypes showed a decrease of 7.3 μg/dL in average cortisol levels if compared to those with GC resistance-genotypes.

**Table 5:** Multivariate analysis for average cortisol compared to baseline levels of SV (A) and SW (B) subjects (n = 27).

Fixed effects	Coefficient	*p-value*
A		
Factor (2-hour mark)	-0.88	0.674
Factor (4-hour mark)	-8.88	0.0064
Age	-0.3	0.0155
GC increased sensitivity-genotype	+7.3	0.0023
B		
Factor (2-hour mark)	+1.64	0.2228
Factor (4-hour mark)	+0.617	0.7270
Age	+0.22	0.011
Fludrocortisone users	+5.3	0.0086

21-OHD: 21-hydroxylase deficiency; GC: glucocorticoid; SW: salt-wasting; SV: simple-virilizing

Most of SW subjects did not present a statistically significant decrease in cortisol levels at the 4-hour time-point of the test. The genotype did not influence average cortisol concentrations among them (p = 0.4872). The final multivariate model included age (p = 0.011) and fludrocortisone-use (p = 0.0086) (**Table 5B**).

Fludrocortisone users had mean cortisol concentrations 5.3 μg/dL higher than non-users. Baseline cortisol concentrations were not statistically different between SV and SW (p = 0.3326, KW), nor between SW fludrocortisone non-users and SV subjects (p = 0.416, KW). However, the levels were lower among SW fludrocortisone users when compared to SW non-users (p = 0.088, KW).

## Discussion

This is a genotype–phenotype association study that revealed pharmacogenomic markers of GC resistance among subjects with classical CAH due to 21-OHD. We found two genotypes of interest that were associated with impaired DST response at 4-hour testing: “*Tth111*I *+ 9β/*Wild”; *“Tth111*I *+ ER22/23EK + 9β/*Wild*”*. Both haplotypes have been associated with relative GC resistance in the literature [5, 8].

The haplotype *Tth111*I *+ ER22/23EK + 9β/*Wild is rarely reported in most populations (~1%), but haplotype-*Tth111*I *+ 9β/*Wild occurs more commonly (~10%) [8, 9, 14]. For diseases other than CAH, GR gene haplotype-phenotype associations studies were performed in various cohorts of children and adults receiving GC treatment: acute lymphoblastic leukaemia [19], cystic fibrosis [20], bowel inflammatory disease [21], psoriasis [22], multiple sclerosis [23], and in Cushing’s [24], metabolic [25] and Guillain Barré [26] syndromes. The studies showed that GR genetic factors associated with GC resistance can affect not only the response to therapy but also disease pathophysiology.

The GR variants influence the expression of molecular properties of the receptor protein, except for *Tth111*I and *Bcl*I, which are located out of protein-coding regions. The *Bcl*I-variant is quite commonly present in the human population. It is located in introns, but the variant may still have important regulatory roles, modifying the expression of mRNA of nearby genes, affecting RNA stability or access of transcription factors to gene regulatory elements [30]. But the exact mechanism by which this polymorphism interferes with GC sensitivity is unclear. It has been widely associated with GC increased sensitivity [27, 28], as we detected in our series among SV subjects, although this variant was less commonly present in our cohort compared to healthy controls [14]. Nevertheless, the homozygosis amplified the effect [29], once the most significant cortisol suppression was observed in the homozygous *Bcl*I-carriers. Importantly, in addition to GC increased response, this variant might play a role in obesity susceptibility among subjects with CAH [11].

The *9β*-polymorphism main actions have been very well-explored in the literature. It is located at the exon-9 of the GR-gene, and it encodes two alternative splicing variants: the α and β isoforms, but only the first one has functional effects. Thus, its related polymorphism has been associated with an increased mRNA expression and stabilization of the dominant-negative splice β-variant [30]. This *9β*-polymorphism was inherited in block with both *ER22* and *23EK* variants in our series [14]. *ER22* and *23EK* variants have been associated to lower body weight, lower total cholesterol, and low-density lipoprotein cholesterol levels, as well as lower fasting insulin concentrations and better insulin sensitivity [31]. They are located on codons 22 and 23 at the transactivation domain of the exon 2. Molecular studies point to a 15% increase in the proportion of the GRα-A translation sub-isoform to GRα-B [32]. All together, these data might explain the physiology of the GC relative increased resistance among subjects of our series.

Herein, regarding the variables which may have affected the GC response, we could highlight the CAH clinical phenotype. SW-participants had consistent less cortisol suppression than SV. Moreover, thirty percent of the SW subjects did not present any cortisol suppression in the DST, all of those exhibited GC resistance-genotypes. Thus, as expected, in our series, some SW-patients had a pattern of greater resistance to GC treatment than others, and this might be, in part, attributed to GR variants.

The fludrocortisone use itself in the eve of test might have had an influence on some of these non-suppressive responses among SW subjects. It is well-known that fludrocortisone crosses the blood-brain barrier and exerts an inhibitory effect on the HPA axis, mainly on serum cortisol levels during the nadir of the circadian rhythm. These effects reflect the complete binding of fludrocortisone to mineralocorticoid receptors (MR) in the hippocampus since there are uncertainties about the occurrence of MRs in the hypothalamus and the pituitary. Finally, it seems that MRs in the hippocampus mediate the "proactive" feedback of GCs, in a dose-response manner, when above 0.05 mg [33–35].

The GC response was also associated with age. Children younger than 10 years old presented more cortisol suppression during the 4-hour test if compared with adolescents and adults. However, this subgroup of children brought together the highest proportion of SV subjects, which may explain this finding, once SV presented more cortisol suppression.

Although our study has some limitations, including a relatively small sample size, which may have biased some results, the rigorous experimental protocol of cortisol measurements at three different time points allowed us to make comparative assessment and data analysis regarding the GC overall response to the DST. To our knowledge, there are no other studies addressing the GR haplotype structure and the GC response to treatment within the CAH population.

In the future, population specific pharmacogenomics landscape relevant for GC therapy would further contribute to better understanding the inconsistency in therapy response and could be helpful in predicting the risk of adverse reactions in those patients receiving GCs [36, 37]. The genetic profiles of GC resistance might also be useful in identifying CAH specific subgroups that would benefit from personalized treatment. Other mechanisms of inherited GC resistance have been identified and should be further explored in CAH subjects [38, 39].

In conclusion, the *Tth111*I *+ 9β/Wild* and *Tth111*I *+ ER22/23EK + 9β/Wild* genotypes are probably pharmacogenomics markers of GC resistance in CAH. These findings may be relevant given the challenges posed by the therapeutic management with GC in CAH and should be confirmed by further studies.

## Supporting information

S1 Data (XLSX)
